# Prospective Environmental Surveillance of *Aspergillus* spp. During Hospital Renovation: Evidence of Potential Fungal Dissemination Through Shared Ceiling Voids

**DOI:** 10.3390/idr18050101

**Published:** 2026-09-14

**Authors:** Mieko Tokano, Norihito Tarumoto

**Affiliations:** 1 Department of Infectious Disease and Infection Control, Saitama Medical University, 38 Morohongo, Moroyama, Saitama 350-0495, Japan; mtokano@saitama-med.ac.jp; 2 Departments of Microbiology, Faculty of Medicine, Saitama Medical University, 38 Morohongo, Moroyama, Saitama 350-0495, Japan

**Keywords:** *Aspergillus fumigatus*, hospital renovation, environmental monitoring, air sampling, healthcare-associated aspergillosis, infection control

## Abstract

Background: Hospital construction and renovation are recognized risk factors for healthcare-associated aspergillosis because demolition work can disperse airborne fungal spores into the air. However, environmental dissemination pathways during renovations remain incompletely understood. Methods: We conducted a prospective environmental surveillance study during the renovation of a high-care unit (HCU) at a 1000-bed tertiary care hospital in Japan. Air sampling was performed before, during, and after the renovation at five locations using an air sampler (500 L/sample). Fungal isolates were identified using internal transcribed spacer sequencing. Dust collected from the ceiling void above the HCU before the renovation was also cultured. Results: *Aspergillus fumigatus* was isolated from dust collected from the ceiling void before renovation. During renovation, *Aspergillus* spp. were detected only in areas sharing the ceiling void with the construction site and on the floor immediately below the renovation area. No *Aspergillus* spp. were detected in the intensive care unit, which did not share the ceiling void. These findings suggest that demolition-related vibrations may have disturbed dust accumulated within the ceiling voids, suggesting a possible mechanism for localized fungal dissemination. Additionally, to assess the clinical impact of the renovation, the quarterly number of patients with positive *Aspergillus*-related microbiological or serological findings from 2015 to 2025 was reviewed. No apparent increase in the number of patients with positive *Aspergillus*-related microbiological or serological findings was observed during the renovation period. Our findings suggest that connected ceiling voids may represent potential pathways for localized fungal dispersal during demolition, while emphasizing that this proposed pathway was not directly confirmed. Conclusions: These findings highlight the importance of environmental surveillance and appropriate infection control measures during hospital renovation.

## 1. Introduction

*Aspergillus* species (spp.) are ubiquitous filamentous fungi that are widely distributed in the environment. Construction, demolition, and renovation activities can aerosolize fungal spores, thereby increasing the risk of their airborne dissemination within healthcare facilities. Numerous studies have demonstrated an association between hospital construction activities and outbreaks of invasive aspergillosis, particularly among immunocompromised patients, such as recipients of hematopoietic stem cell transplantation or patients with hematological malignancies [1,2,3,4].

To minimize this risk, the Centers for Disease Control and Prevention (CDC) recommends implementing infection prevention measures based on an Infection Control Risk Assessment (ICRA) before initiating construction or renovation projects. ICRA provides a systematic framework for evaluating construction-related risks according to the type of work, patient population at risk, and required engineering and administrative controls, including dust containment, negative-pressure barriers, high-efficiency particulate air (HEPA) filtration, and ventilation management [5].

Despite these recommendations, evidence regarding the actual dynamics of airborne *Aspergillus* during hospital renovations remains limited. Furthermore, recent investigations of a healthcare-associated *Aspergillus* spp. cluster in an Australian transplant unit have renewed attention to the importance of environmental monitoring during construction, highlighting deficiencies in fungal surveillance and construction-related infection control as contributing factors identified by the official investigation [6].

Our hospital underwent a large-scale renovation of the High Care Unit (HCU), involving extensive demolition work that was classified as the highest-risk (Class V) construction project according to the Association for the Healthcare Environment’s Infection Control Risk Assessment [7]. To evaluate whether our infection prevention measures were adequate, we prospectively performed environmental air sampling before, during, and after renovation at multiple locations within the hospital and assessed whether renovation was associated with changes in airborne *Aspergillus* contamination or an increase in the number of aspergillosis cases.

## 2. Materials and Methods

### 2.1. Study Setting and Design

This study was a prospective environmental surveillance study with a retrospective review of microbiological records conducted at Saitama Medical University Hospital, a tertiary care teaching hospital with approximately 1000 beds located in Saitama, Japan. A major renovation of the HCU was performed between 30 September 2023 and 29 February 2024. Environmental air sampling was prospectively performed before, during, and after the renovation to evaluate changes in airborne fungal contamination. In addition, microbiological records from January 2015 to December 2025 were retrospectively reviewed to assess whether the renovation period was associated with an increase in patients with microbiological evidence of aspergillosis. At the time of renovation, the hospital building was 42 years old. Because the project involved extensive demolition work over a prolonged period, the renovation was classified as a Class V project, representing the highest level of infection risk according to the ASHE’s Infection Control Risk Assessment [7]. Infection prevention measures were implemented based on these guidelines during the renovation period. In addition, all hospital wards were instructed to keep the windows closed to minimize the entry of airborne fungal spores from the outdoor environment. The HCU is located on the seventh floor of the South Building. The intensive care unit (ICU) located on the same floor is structurally independent and does not share the ceiling void with the HCU. The ICU had an independent ventilation system and was maintained under negative pressure, which was routinely confirmed by smoke testing. The other areas included in the environmental sampling were maintained under neutral pressure. In contrast, the seventh floor of the Main Building is connected to the South Building and shares the same ceiling void as the HCU renovation area.

### 2.2. Study Design and Environmental Air Sampling

**Figure 1:**
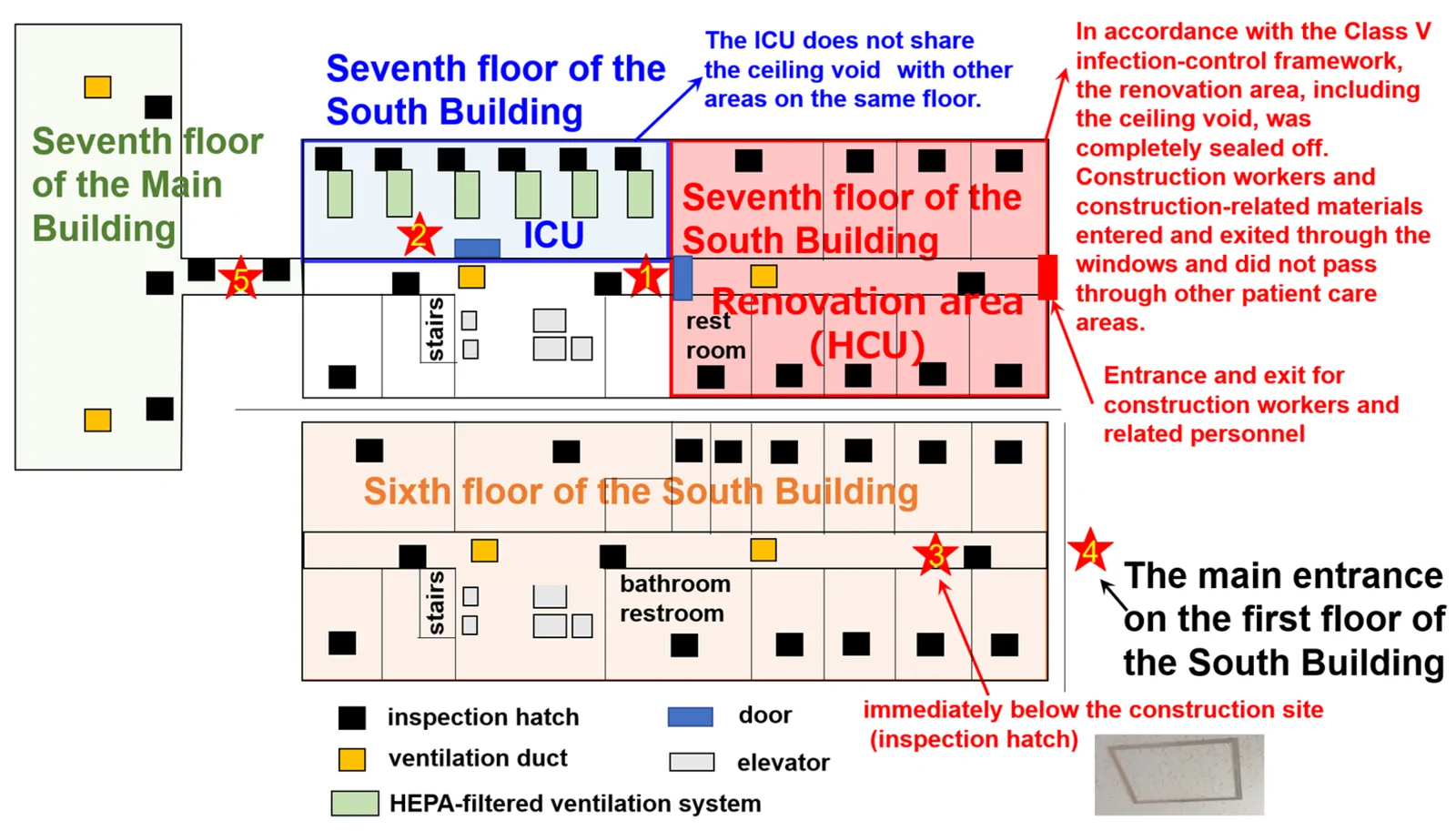
Locations of air sampling sites during hospital renovation. The star symbols (★) indicate the sampling locations. The numbers correspond to those provided in the main text.

Environmental air sampling was performed before, during, and after renovation to evaluate the impact of renovation work on airborne fungal contamination. Sampling was conducted at five locations: (1) the entrance to the HCU, (2) the ICU, (3) the sixth floor of the South Building immediately below the renovation site (inspection hatch), (4) the main entrance on the first floor of the South Building, and (5) the seventh floor of the Main Building (Figure 1). Air samples were collected using an ES-100 air sampler (Sysmex, Kobe, Japan) equipped with potato dextrose agar plates (Becton Dickinson, Sparks, MD, USA). A total volume of 500 L of air was sampled at each site (100 L/min for 5 min), consistent with previously published hospital environmental surveillance studies using volumetric air sampling for airborne *Aspergillus* detection [8]. After sampling, the plates were incubated at 35 °C for 7 days, and fungal colonies were counted and identified.

Air sampling was performed at five locations before, during, and after the renovation of the high care unit (HCU): (1) the entrance to the HCU, (2) the intensive care unit (ICU), which did not share the ceiling void with the renovation area, (3) the sixth floor of the South Building directly beneath the renovation site, (4) the first-floor entrance of the South Building, and (5) the seventh floor of the Main Building, which shared the ceiling void with the renovation area. The renovation site (HCU) is marked in red on the map.

### 2.3. Identification of Aspergillus Isolates

Colonies morphologically suspected to be *Aspergillus* spp. were subcultured in Sabouraud liquid medium at 35 °C for 2–3 days and subsequently incubated at room temperature with gentle agitation for several days to promote fungal growth. Genomic DNA was extracted after bead-beating using the DNeasy Plant Mini Kit (QIAGEN, Hilden, Germany). Molecular identification was performed using PCR amplification and Sanger sequencing of the internal transcribed spacer (ITS) region. The primer sequences were ITS1 (5′-TCCGTAGGTGAACCTGCGG-3′) and ITS4 (5′-TCCTCCGCTTATTGATATGC-3′). PCR amplification of the ITS region was performed using *A. fumigatus* reference strain MYA-3626 as a positive control and phosphate-buffered saline (PBS) as a negative control. The negative control was included in each PCR run to monitor potential contamination. Species identification was performed by comparison with reference ITS sequences in public nucleotide databases (BLAST). Following previous studies [9], an ITS sequence similarity of ≥97% was used as the criterion for species-level identification. Species identification was performed by comparison with reference ITS sequences in public nucleotide databases. Because the primary objective was to confirm the presence of *Aspergillus* spp. rather than to achieve definitive species-level identification within closely related *Aspergillus* species complexes, additional sequencing of secondary loci was not performed.

### 2.4. Pre-Renovation Environmental Assessment

Before the renovation, dust samples were collected from the ceiling void through an inspection hatch located above the HCU to evaluate the baseline fungal contamination. Dust was collected from a single location within the ceiling void using a sterile sampling device (sterile swab (Hakuzo Medical Corporation, Osaka, Japan)/sterile container (Corning, NY, USA)), and the collected material was subjected to mycological culture. This area was selected because demolition-related vibrations were expected to dislodge accumulated dust into the clinical environment. Dust samples were cultured, and fungal isolates were identified using the same methods described in Section 2.3. One colony of *Aspergillus fumigatus* was isolated before the renovation commenced.

### 2.5. Antifungal Susceptibility Testing

Antifungal susceptibility testing was performed for three *A. fumigatus* isolates identified by ITS sequencing according to the Clinical and Laboratory Standards Institute (CLSI) M38, 3rd edition, reference broth microdilution method. Among the antifungal agents tested, voriconazole (VRCZ) was the only agent for which applicable CLSI epidemiological cutoff values and clinical breakpoints were available in this document. Azole resistance was interpreted using the current CLSI epidemiological cutoff values and clinical breakpoints, where applicable.

### 2.6. Evaluation of Aspergillosis Cases

To investigate whether renovation work was associated with an increased incidence of aspergillosis, microbiological records from January 2015 to December 2025 were retrospectively reviewed. Patients were screened for positive *Aspergillus*-related microbiological or serological findings based on the following predefined criteria: Patients were included if they met at least one of the following microbiological criteria: (1) positive serum *Aspergillus* galactomannan antigen; (2) positive anti-*Aspergillus* antibody; or (3) isolation of *Aspergillus* spp. from respiratory specimens, including sputum, bronchoalveolar lavage fluid, or pleural fluid. These findings were used for descriptive environmental surveillance and were not intended to establish a diagnosis of proven, probable, or possible aspergillosis according to the EORTC/MSGERC criteria. A galactomannan index of ≥1.0 was considered positive. Until October 2022, anti-*Aspergillus* precipitating antibody testing was performed, whereas from November 2022, anti-*Aspergillus* IgG was measured. An IgG concentration of >10 AU/mL was considered positive. The quarterly number of microbiologically positive patients was compared to determine whether an outbreak of aspergillosis occurred during the renovation period.

## 3. Results

### 3.1. Environmental Air Sampling

The weather conditions, ambient temperature, and relative humidity at each sampling point are summarized in Table 1.

The sampling results are shown in Table 2. Because 500 L (0.5 m^3^) of air was sampled, one detected colony corresponded to 2 CFU/m^3^. Air sampling at the main entrance on the first floor of the South Building consistently detected environmental fungi, including *Aspergillus* and *Penicillium* spp., throughout the study period (Table 2). No apparent increase in the recovery of these fungi was observed during the renovation period compared to the pre-renovation or post-renovation periods. In contrast, indoor sampling demonstrated an increased detection of *Aspergillus* spp. during the renovation period (Table 2). *Aspergillus* spp. were isolated from the seventh floor of the Main Building, which shared the ceiling void with the renovated HCU, and from the sixth floor of the South Building, which was located immediately beneath the renovation site. *Aspergillus* spp. were also recovered during the renovation of the HCU entrance. No *Aspergillus* spp. were detected in samples collected from the ICU, which did not share the ceiling space with the renovation area, even though air sampling was performed at a location remote from the HEPA-filtered air supply outlet. The fungal species identified at each sampling site are summarized in Table 2. Three isolates identified as *A. fumigatus* by internal transcribed spacer (ITS) sequencing were subjected to antifungal susceptibility testing. As shown in Table 2, two of these isolates were recovered from the seventh floor of the Main Building, and one was recovered from the sixth floor of the South Building. Antifungal susceptibility testing was performed using the standardized broth microdilution method described in the Clinical and Laboratory Standards Institute (CLSI) M38, 3rd edition, a widely used international reference standard. The MICs (minimum inhibitory concentrations) of VRCZ were 0.5 μg/mL for one isolate and 0.25 μg/mL for the other two isolates, and none of the isolates exhibited azole resistance.

### 3.2. Clinical Surveillance for Aspergillosis

**Figure 2:**
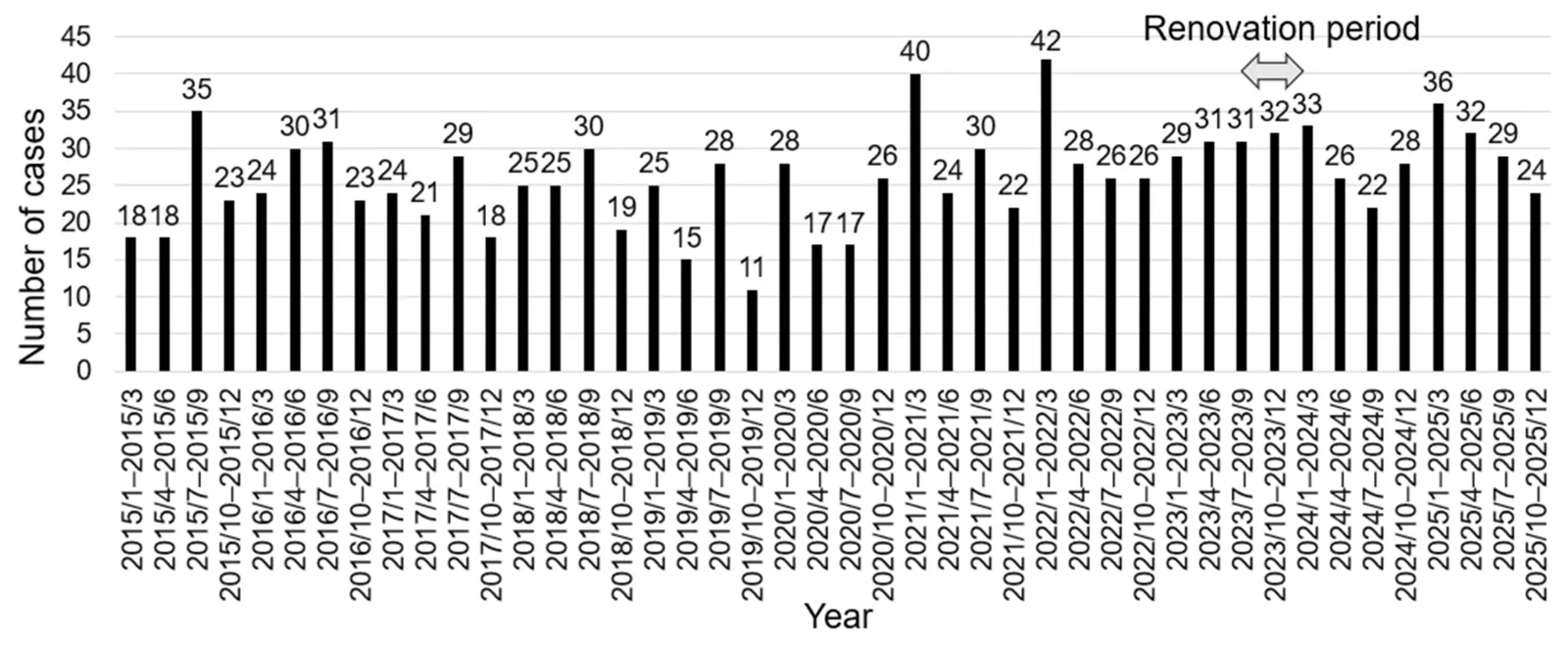
Quarterly number of patients with positive *Aspergillus*-related microbiological or serological findings from 2015 to 2025. No specific interventions were undertaken in response to positive environmental samples because there was no increase in the number of patients with positive findings.

The quarterly number of patients with positive *Aspergillus*-related microbiological or serological findings, defined as at least one of the following: positive serum galactomannan antigen, positive anti-*Aspergillus* antibody, or isolation of *Aspergillus* spp. from respiratory specimens, is shown in Figure 2. No apparent increase in the number of microbiologically positive patients was observed during the renovation period. Similarly, no outbreaks of pulmonary aspergillosis were identified during or immediately after the renovation period.

The quarterly number of patients with at least one positive *Aspergillus*-related test result is shown. Patients were included if they had a positive galactomannan antigen test (cutoff index ≥ 1.0), a positive anti-*Aspergillus* antibody test (precipitating antibody until October 2022 or *Aspergillus*-specific IgG > 10 AU/mL from November 2022 onward), or a respiratory specimen positive for *Aspergillus* spp. by culture. The arrows indicate the hospital renovation period (September 2023–February 2024). No apparent increase in the number of patients with positive *Aspergillus*-related microbiological or serological findings was observed during the renovation period.

## 4. Discussion

This prospective environmental surveillance study revealed several critical findings regarding the dissemination of *Aspergillus* spp. during hospital renovation. The primary novelty of our study is the suggestion that the ceiling void may represent a potential reservoir and pathway for fungal dissemination. While it is well established that renovation activities generate aerosolized dust [1], our evidence shows that *Aspergillus* was isolated from the ceiling void prior to construction and subsequently detected exclusively in areas sharing that specific structural void. These findings suggest that the shared ceiling void may have served as a potential local reservoir or pathway for fungal dissemination during the demolition. We hypothesized that mechanical vibrations caused by demolition work may have disturbed the accumulated dust and contributed to the release of fungal spores into the clinical environment. However, this proposed mechanism was not directly evaluated in the present study.

Furthermore, our findings revealed a significant vertical dissemination pattern, as *Aspergillus* spp. were detected on the floor immediately below the renovation site. We hypothesized that mechanical vibrations from demolition work propagated through the building’s structure, disturbing accumulated dust in the ceiling void of the floor below, and releasing spores into the clinical environment. This suggests that fungal exposure may extend vertically through structural connections, not just horizontally. However, direct measurements of vibration, dust movement, or airflow were not performed, and this proposed mechanism therefore remains hypothetical.

In contrast, the effectiveness of structural isolation and HEPA-filtered ventilation was underscored by the total absence of *Aspergillus* spp. in the ICU, despite its proximity to the renovation area. The ICU was routinely confirmed to have negative airflow by smoke testing. The lack of a shared ceiling void and independent ventilation system in the ICU may have contributed to the absence of detectable contamination.

Finally, an important finding of this study was that an increase in the environmental detection of *Aspergillus* spp. did not necessarily correspond to adverse clinical outcomes in the patients. Consistent with previous reports [1], despite a transient increase in environmental *Aspergillus* detection, no increase in patients with positive *Aspergillus*-related microbiological or serological findings or outbreaks was observed. This indicates that comprehensive infection prevention measures based on the ICRA, including Class V precautions, dust barriers, and environmental cleaning, may have contributed to mitigating the risk to patients. Consequently, environmental surveillance should be regarded as a complementary tool for evaluating control measures rather than a direct predictor of clinical infection. Based on these insights, we recommend that high-risk patients be accommodated in wards that are not only horizontally distant but also structurally and vertically isolated from the renovation activities.

This study had several limitations. First, the number and frequency of environmental sampling sessions were limited, and therefore, temporal fluctuations in airborne fungal contamination throughout the prolonged renovation period may not have been fully captured. The number of environmental samples was also limited, precluding robust statistical comparisons of fungal recovery between the pre-, during-, and post-renovation periods. Baseline dust sampling was performed at only one location within the ceiling void, and replicate sampling was not performed. Therefore, the detected fungal contamination may not represent the overall fungal burden within the ceiling void. Environmental fungal burden was assessed by culture, and continuous quantitative monitoring of airborne fungal concentrations was not performed. Additional environmental sampling after completion of the renovation was not feasible because of budgetary constraints. More frequent and continuous environmental monitoring would be desirable in future studies to better characterize temporal changes in airborne fungal contamination. In addition, the number of environmental sampling sessions was limited, and the possibility that seasonal variations in temperature and humidity influenced fungal recovery cannot be excluded from consideration. However, previous studies have reported that although the concentrations of airborne fungi other than *Aspergillus* spp. tend to increase during the summer months, airborne *Aspergillus* spp. exhibit little seasonal variation [8]. Therefore, we consider it unlikely that seasonal factors had a substantial impact on the detection of *Aspergillus* spp. in this study. Second, although fungal species were identified by ITS sequencing, ITS sequencing was considered sufficient to confirm the presence of *Aspergillus* spp. for the purpose of this study, but it may not provide definitive species-level discrimination within certain *Aspergillus* species complexes. Furthermore, the strain-level genetic relatedness between environmental and clinical isolates was not investigated. Whole-genome sequencing or microsatellite typing would provide stronger evidence regarding the source of fungal dissemination. Third, the environmental fungal burden was evaluated qualitatively by culture, and airborne spore concentrations were not quantified using molecular methods or continuous air monitoring. Fourth, the quarterly number of patients with positive *Aspergillus*-related microbiological or serological findings may have been influenced by changes in hospital activity, patient population, and diagnostic practices, including the change in the anti-*Aspergillus* antibody testing method in November 2022. Therefore, these quarterly counts should be interpreted as descriptive surveillance data rather than measures of disease incidence.

Finally, this was a single-center study, which may limit the generalizability of our findings to other centers.

## 5. Conclusions

In conclusion, our findings suggest that shared ceiling voids may represent an important potential pathway for localized dissemination of *Aspergillus* spp. during hospital renovation. However, this proposed pathway should be interpreted cautiously, as direct measurements of dust movement, airflow dynamics, and strain-level relatedness were not performed. Careful environmental surveillance together with ICRA-based infection prevention measures may help minimize the risk of healthcare-associated aspergillosis during construction activities.

## Figures and Tables

**Table 1:** Environmental conditions at the time of air sampling.

		Before Renovation (22 September 2023)	During Renovation (10 November 2023)	After Renovation (11 March 2024)
Weather conditions		Cloudy	Sunny	Sunny
Indoor	ambient temperature	24.2–25.8 °C	19.8–23.0 °C	18.2–21.9 °C
	relative humidity	59–67%	52–57%	22–24%
Outdoor	ambient temperature	25.6 °C	24.3 °C	20.5 °C
	relative humidity	72%	43%	22%

**Table 2:** Fungal species recovered by air sampling before, during, and after hospital renovation.

		Before Renovation (CFU/m^3^)	During Renovation (CFU/m^3^)	After Renovation (CFU/m^3^)
Outdoor	*Aspergillus fumigatus*	2		
	*Aspergillus niger*	2		2
	*Aspergillus* spp.		2	
	*Penicillium citrinum*			2
	*Penicillium* spp.		2	
	*Byssochlamys spectabilis*	2		
	*Trichoderma reesei*		2	
Indoor	*Aspergillus fumigatus*		4 (Seventh floor of the Main Building)	2 (Sixth floor of the South Building)
	*Aspergillus niger*		2 (Seventh floor of the Main Building)	
	*Aspergillus nidulans*	2 (Seventh floor of the Main Building)		
	*Aspergillus* spp.		2 (Sixth floor of the South Building)	
	*Penicillium cuddlyae*	2 (Seventh floor of the South Building)		
	*Penicillium oxalicum*	4 (Sixth floor of the South Building)		
	*Trametes hirsuta*	2 (Seventh floor of the South Building)		
	*Curvularia* spp.	2 (Seventh floor of the South Building)		
	*Schizophyllum commune*	2 (Sixth floor of the South Building)		
	*Arthrographis kalrae*			2 (Seventh floor of the Main Building)
	*Choanephora cucurbitarum*	2 (Seventh floor of the Main Building)		
	Unidentified species		2 (Seventh floor of the South Building)	

## Data Availability

The original contributions presented in this study are included in the article. Further inquiries can be directed to the corresponding author.

## Abbreviations

The following abbreviations are used in this manuscript:

CDC	Centers for Disease Control and Prevention
ICRA	Infection Control Risk Assessment
HEPA	high-efficiency particulate air
HCU	High Care Unit
ICU	Intensive care unit
ITS	internal transcribed spacer
VRCZ	voriconazole
